# Crystal, mol­ecular structure and Hirshheld surface analysis of 5-hy­droxy-3,6,7,8-tetra­meth­oxy­flavone

**DOI:** 10.1107/S2056989020013596

**Published:** 2020-10-13

**Authors:** Haji Akber Aisa, Lidiya Izotova, Abdurashid Karimov, Erkin Botirov, Azimjon Mamadrahimov, Bahtiyar Ibragimov

**Affiliations:** a Key Laboratory of Plants Resources and Chemistry of Arid Zone, Xinjiang, Technical Institute of Physics and Chemistry, Chinese Academy of Science, Urumqi 830011, People’s Republic of China; b Institute of Bioorganic Chemistry, UzAS, M. Ulugbek Str., 83, 100125,Tashkent, Uzbekistan; c Institute of the Chemistry of Plant Substances, UzAS, M. Ulugbek Str., 77, 100170, Tashkent, Uzbekistan

**Keywords:** crystal structure, flavones, Hirshfeld surface analysis, hydrogen bonding

## Abstract

The title compound (systematic name: 5-hydroxy-3,6,7,8-tetramethoxy-2-phenyl-4*H*-chromen-4-one), C_19_H_18_O_7_, is a flavone that was isolated from a butanol extract of the herb *Scutellaria nepetoides M. Pop*. The flavone mol­ecule is almost planar, with a dihedral angle between the planes of the benzo­pyran-4-one group and the attached phenyl ring of 6.4 (4)°. The 5-hy­droxy group forms a strong intra­molecular hydrogen bond with the carbonyl group, resulting in a six-membered hydrogen-bonded ring. The crystal structure has triclinic (*P* ) symmetry. In the crystal, the mol­ecules are linked by C—H⋯O hydrogen bonds into a two dimensional network parallel to the *ab* plane. The Hirshfeld surface analysis indicates that the most important contributions to the crystal packing are from H⋯H (53.9%) and H⋯O/O⋯H (20.9%) inter­actions.

## Abstract 2

5-Hy­droxy-3,6,7,8-tetra­meth­oxy­flavone was isolated from a butanol extract of the herb *Scutellaria nepetoides M. Pop*. and its structure has been established by X-ray crystallographic analysis.

## Chemical context   

Flavonoids are the most numerous class of natural phenolic compounds, which are characterized by structural diversity, high and versatile activity and low toxicity. Plants of the genus *Scutellaria L*. are widespread in Europe, North America, East Asia and are extensively used in traditional Chinese medicine (Shang *et al.*, 2010 ▸). Flavonoids isolated from plants of the genus *Scutellaria L.* exhibit anti­tumor (Yu *et al.*, 2007 ▸), hepatoprotective (Jang *et al.*, 2003 ▸), anti­oxidant (Sauvage *et al.*, 2010 ▸), anti-inflammatory (Dai *et al.*, 2013 ▸), anti­convulsant (Park *et al.*, 2007 ▸), anti­microbial (Arituluk *et al.*, 2019 ▸) and anti­viral activity (Leonova *et al.*, 2020 ▸). The creation of drugs based on flavonoids is based on the establishment of the ‘chemical structure–pharmacological properties’ relationship, and the determination of the structure of a new flavonoid may become a key starting point.
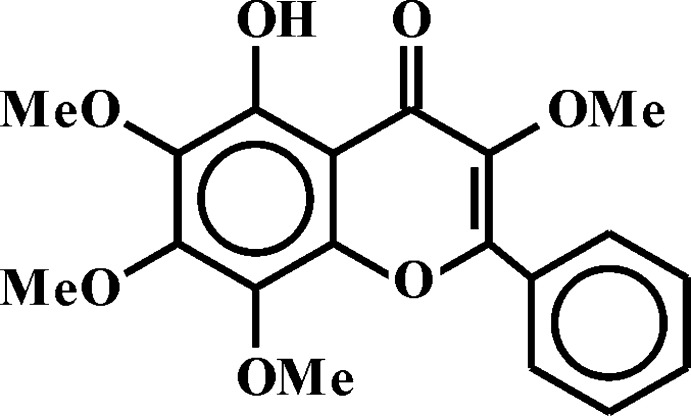


## Structural commentary   

**Figure 1:**
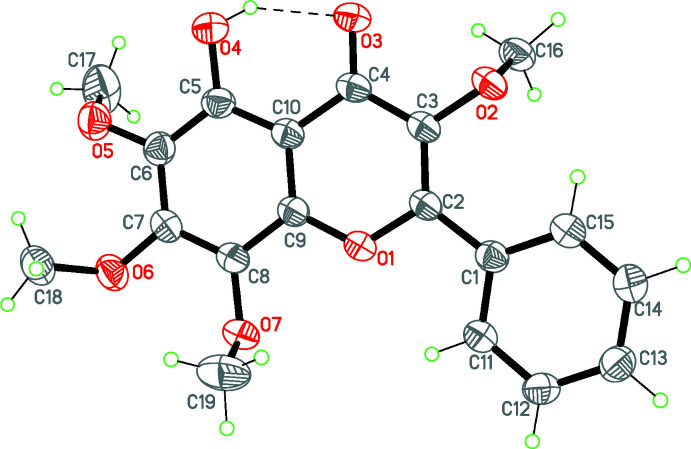
The mol­ecular structure of the title compound with the atom labelling. Displacement ellipsoids are drawn at the 50% probability level.

The mol­ecular structure of the title compound is presented in Fig. 1 ▸. The benzo­pyran moieties are practically planar, with r.m.s. deviations of 0.01 Å. The mol­ecular conformation is restricted by the relative positions of the benzo­pyran unit and the phenyl ring, the dihedral angle between them being 6.4 (4)°. Atoms C3, C6, C7 and C8 of the meth­oxy substituent have an out-of-plane conformation with the meth­oxy groups at atoms C3 and C6 pointing in the same direction [C16—O2—C3—C2 = 109.3 (2) and C17—O5—C6—C5 = 66. 7(4)°], while the meth­oxy groups at atoms C7 and C8 point in opposite direction [C18—O6—C7—C6 = −56.3 (3) and C19—O7—C8—C7 = −91.4 (3)°]. The conformation of the mol­ecule is fixed because of the intra­molecular O4—H4⋯O3 hydrogen bond [2.599 (2) Å, 147°], which closes a six-membered ring with graph-set notation *S*(6) (Etter, 1990 ▸).

## Supra­molecular features   

**Figure 2:**
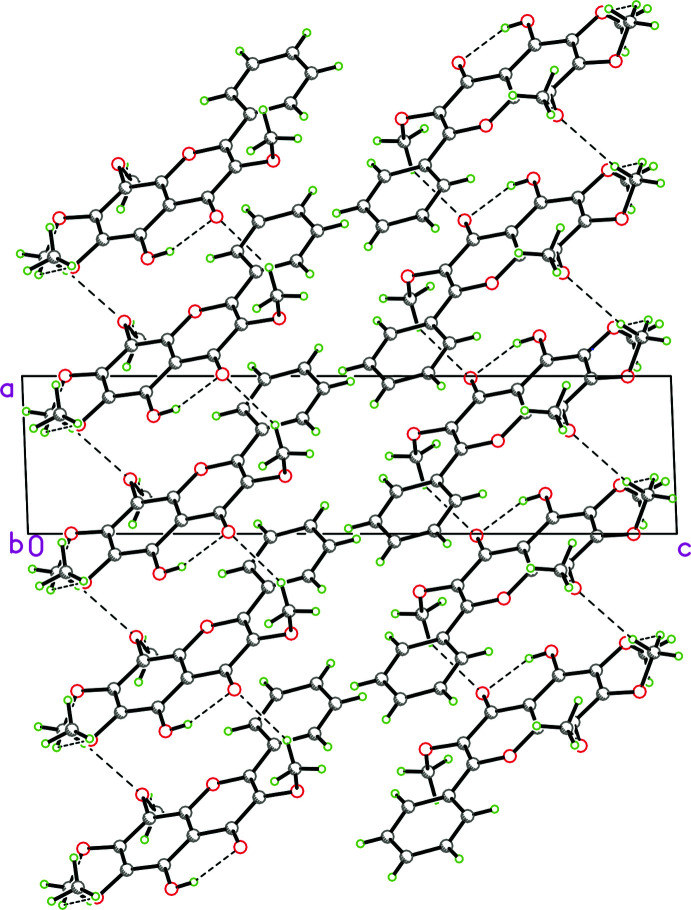
Crystal structure of the title compound in projection on the *ac* plane. Hydrogen bonds are shown as dashed lines.

In the crystal, the mol­ecules are linked by C—H⋯O hydrogen bonds into a two dimensional network parallel to the *ab* plane. A perspective view of the crystal packing in the unit cell is depicted in Fig. 2 ▸ and numerical details of the hydrogen bonds are presented in Table 1 ▸.

## Hirshfeld surface analysis   

**Figure 3:**
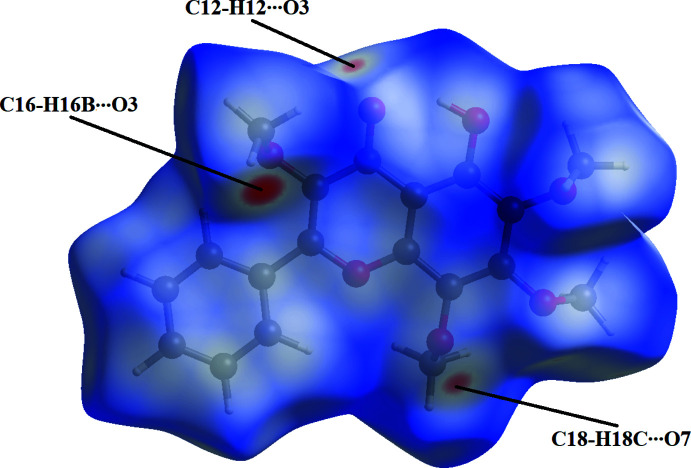
The Hirshfeld surface analysis indicates that the most important contributions to the crystal packing are from H⋯H (53.9%) and H⋯O/O⋯H (20.9%) inter­actions.

**Figure 4:**
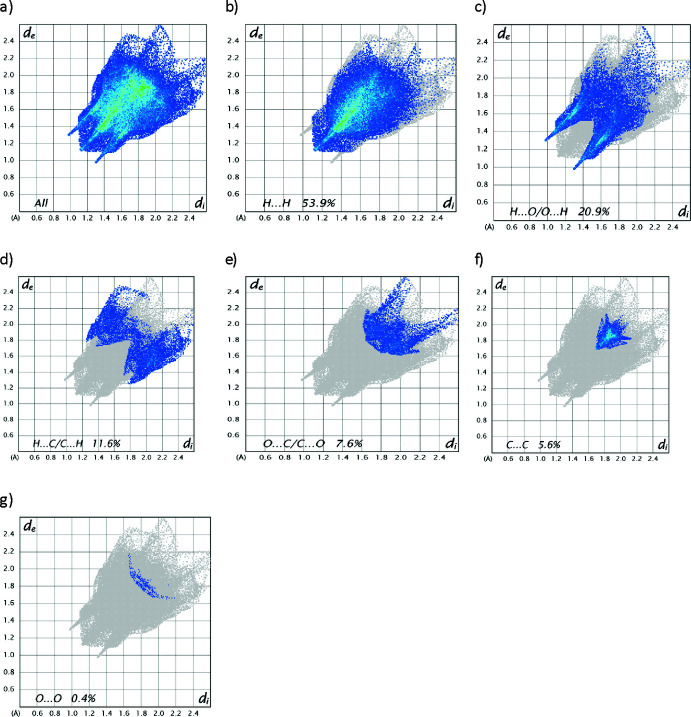
Full two-dimensional fingerprint plots for the title compound, showing all inter­actions (*a*), and delineated into (*b*) H⋯H, (*c*) H⋯O/O⋯H, (*d*) H⋯C/C⋯H, (*e*) O⋯C/C⋯O, (*f*) C⋯C and (*g*) O⋯O inter­actions. The *d*_i_ and *d*_e_ values are the closest inter­nal and external distances (in Å) from a given point on the Hirshfeld surface.

In order to visualize the inter­molecular inter­actions in the crystals of the title compound, a Hirshfeld surface analysis was carried out using *Crystal Explorer 17.5* (Turner *et al.*, 2017 ▸). The Hirshfeld surface mapped over *d*_norm_ (Fig. 3 ▸) shows the expected bright-red spots near atoms O3, O7, H16*B*, which are involved in the C—H⋯O hydrogen-bonding inter­actions. Fingerprint plots (Fig. 4 ▸) reveal that H⋯H and H⋯O/O⋯H inter­actions make the greatest contributions to the surface contacts, while H⋯C/C⋯H, O⋯C/C⋯O, C⋯C and O⋯O contacts are less significant.

## Database survey   

A search of the Cambridge Structural Database (CSD Version 5.41, update of November 2019; Groom *et al.*, 2016 ▸) found 311 hits for the term ‘flavones’. Among these, nine are tetra­meth­oxy­flavones: 3,4′,6,7 (DAVREN; Geng *et al.*, 2011 ▸), 6,2′3′,4′- (JEMGIN; Wallet *et al.*, 1990*a* ▸) and 2′,4′,5,7- (KEPLEW; Wallet *et al.*, 1990*b* ▸), 3,4′,6,7- (MENSII; Meng *et al.*, 2006 ▸), 3′,4′,5,7- (PIQPEK; Shoja, 1997 ▸), 3,4′5,7- (PUGKEI; Aree *et al.*, 2009 ▸), 3′,5,5′,6- (TMOFLV10; Ting *et al.*, 1972 ▸), 3,7,4′,5′- (YASCIF; Etti *et al.*, 2005 ▸). The compound FATZOR (Vijayalakshmi *et al.*, 1986 ▸) is also a 3,6,7,8 tetra­methyl­flavone, but with two hy­droxy substituents at the 5,4′-positions.

## Synthesis and crystallization   

Air-dried whole plants (1.1 kg) of *Scutellaria nepetoides M. Pop*. were extracted three times (each 3 h) with butanol (5 l) at 353 K. The butanol filtrates were collected and concentrated under reduced pressure to provide 10.2 g of butanol extract. The butanol extract (1 g) was subjected to silica gel (60–100 mesh) column (gradient of butanol:water = 0:1, 2:8, 1:1, 8:2, 1:0) as eluent, and five fractions were collected according to TLC analysis. All fractions were concentrated under reduced pressure. A crystallization procedure with different solvents at high temperature was used to obtain the pure compounds. Fraction 5 (0.23 g) was eluted with butanol (100%) at 353 K and with ethanol (95%) at 343 K. The obtained polycrystals were removed from the butanol solution by filtration. Yellow prismatic single crystals were prepared by slow evaporation of butanol solution at room temperature.

## Refinement   

Crystal data, data collection and structure refinement details are summarized in Table 2 ▸. The C-bound H atoms were positioned geometrically and were included in the refinement in the riding-model approximation, with C—H = 0.96 Å (CH_3_), 0.93 Å (aryl H) and O—H = 0.82 Å and with *U*_iso_(H) = 1.2*U*_eq_(C) (aryl H) and 1.5*U*_eq_(C-methyl, O).

## Supplementary Material

Crystal structure: contains datablock(s) I. DOI: 10.1107/S2056989020013596/zn2001sup1.cif

Structure factors: contains datablock(s) I. DOI: 10.1107/S2056989020013596/zn2001Isup2.hkl

Supporting information file. DOI: 10.1107/S2056989020013596/zn2001Isup3.cml

CCDC reference: 2036551

Additional supporting information:  crystallographic information; 3D view; checkCIF report

## Figures and Tables

**Table 1:** Hydrogen-bond geometry (Å, °)

*D*—H⋯*A*	*D*—H	H⋯*A*	*D*⋯*A*	*D*—H⋯*A*
O4—H4⋯O3	0.82	1.87	2.599 (2)	147
C16—H16*A*⋯O3	0.96	2.51	3.079 (3)	118
C16—H16*B*⋯O3^i^	0.96	2.39	3.258 (3)	150
C18—H18*B*⋯O5	0.96	2.28	2.897 (4)	121
C18—H18*C*⋯O7^ii^	0.96	2.53	3.278 (4)	135
C17—H17*C*⋯O4	0.96	2.52	3.010 (4)	111

Symmetry codes: (i) 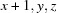; (ii) 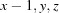.

**Table 2:** Experimental details

Crystal data	
Chemical formula	C_19_H_18_O_7_
*M*_r_	358.33
Crystal system, space group	Triclinic, *P* 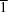
Temperature (K)	293
*a*, *b*, *c* (Å)	5.0789 (4), 8.0801 (6), 20.8682 (19)
α, β, γ (°)	92.481 (7), 91.984 (7), 94.253 (6)
*V* (Å^3^)	852.62 (12)
*Z*	2
Radiation type	Cu *K*α
μ (mm^−1^)	0.90
Crystal size (mm)	0.03 × 0.02 × 0.01

Data collection	
Diffractometer	Agilent Xcalibur, Ruby
Absorption correction	Multi-scan (*CrysAlis PRO*; Agilent, 2014 ▸)
*T*_min_, *T*_max_	0.818, 1.000
No. of measured, independent and observed [*I* > 2σ(*I*)] reflections	6484, 3458, 2408
*R*_int_	0.023
(sin θ/λ)_max_ (Å^−1^)	0.630

Refinement	
*R*[*F* ^2^ > 2σ(*F* ^2^)], *wR*(*F* ^2^), *S*	0.056, 0.174, 1.03
No. of reflections	3458
No. of parameters	240
H-atom treatment	H-atom parameters constrained
Δρ_max_, Δρ_min_ (e Å^−3^)	0.22, −0.18

Computer programs: *CrysAlis PRO* (Agilent, 2014 ▸), *XP* (Siemens, 1994 ▸), *SHELXT2018/2* (Sheldrick, 2015*a* ▸) and *SHELXL2018/3* (Sheldrick, 2015*b* ▸).

## supplementary crystallographic information

### Crystal data

**Table:**

C_19_H_18_O_7_	*Z* = 2
*M**_r_* = 358.33	*F*(000) = 376
Triclinic, *P*1	*D*_x_ = 1.396 Mg m^−^^3^
*a* = 5.0789 (4) Å	Cu *K*α radiation, λ = 1.54184 Å
*b* = 8.0801 (6) Å	Cell parameters from 1498 reflections
*c* = 20.8682 (19) Å	θ = 5.5–75.0°
α = 92.481 (7)°	µ = 0.90 mm^−^^1^
β = 91.984 (7)°	*T* = 293 K
γ = 94.253 (6)°	Prism, yellow
*V* = 852.62 (12) Å^3^	0.03 × 0.02 × 0.01 mm

### Data collection

**Table:**

Agilent Xcalibur, Ruby diffractometer	*R*_int_ = 0.023
Radiation source: Enhance (Cu) X-ray Source	θ_max_ = 76.1°, θ_min_ = 4.2°
/ω scans	*h* = −6→4
Absorption correction: multi-scan (CrysAlisPro; Agilent, 2014)	*k* = −10→9
*T*_min_ = 0.818, *T*_max_ = 1.000	*l* = −25→25
6484 measured reflections	3 standard reflections every 100 reflections
3458 independent reflections	intensity decay: 2.6%
2408 reflections with *I* > 2σ(*I*)	

### Refinement

**Table:**

Refinement on *F*^2^	0 restraints
Least-squares matrix: full	Hydrogen site location: inferred from neighbouring sites
*R*[*F*^2^ > 2σ(*F*^2^)] = 0.056	H-atom parameters constrained
*wR*(*F*^2^) = 0.174	*w* = 1/[σ^2^(*F*_o_^2^) + (0.0902*P*)^2^ + 0.0534*P*] where *P* = (*F*_o_^2^ + 2*F*_c_^2^)/3
*S* = 1.03	(Δ/σ)_max_ < 0.001
3458 reflections	Δρ_max_ = 0.22 e Å^−^^3^
240 parameters	Δρ_min_ = −0.18 e Å^−^^3^

### Special details

**Table:**

Geometry. All esds (except the esd in the dihedral angle between two l.s. planes) are estimated using the full covariance matrix. The cell esds are taken into account individually in the estimation of esds in distances, angles and torsion angles; correlations between esds in cell parameters are only used when they are defined by crystal symmetry. An approximate (isotropic) treatment of cell esds is used for estimating esds involving l.s. planes.

### Fractional atomic coordinates and isotropic or equivalent isotropic displacement parameters (Å^2^)

**Table:**

	*x*	*y*	*z*	*U*_iso_*/*U*_eq_	
O1	0.4105 (3)	0.28804 (17)	0.27044 (7)	0.0575 (4)	
O2	0.3672 (3)	0.59876 (19)	0.39973 (8)	0.0659 (4)	
O7	0.3450 (3)	0.10219 (19)	0.16067 (8)	0.0685 (4)	
O3	0.0122 (3)	0.69625 (19)	0.30889 (9)	0.0714 (5)	
O4	−0.2527 (4)	0.6406 (2)	0.20033 (9)	0.0740 (5)	
H4	−0.209283	0.687910	0.235051	0.111*	
O6	0.0019 (3)	0.1855 (2)	0.06667 (8)	0.0725 (5)	
O5	−0.3071 (4)	0.4564 (2)	0.08488 (9)	0.0792 (5)	
C9	0.2302 (4)	0.3341 (2)	0.22626 (10)	0.0532 (5)	
C2	0.4615 (4)	0.3764 (2)	0.32748 (10)	0.0531 (5)	
C1	0.6627 (4)	0.3000 (2)	0.36663 (10)	0.0547 (5)	
C3	0.3289 (4)	0.5145 (2)	0.34102 (10)	0.0559 (5)	
C10	0.0862 (4)	0.4721 (2)	0.23720 (11)	0.0549 (5)	
C8	0.1951 (4)	0.2349 (3)	0.17031 (11)	0.0570 (5)	
C4	0.1327 (4)	0.5696 (3)	0.29666 (11)	0.0576 (5)	
C5	−0.1022 (4)	0.5106 (3)	0.19027 (12)	0.0598 (5)	
C7	0.0153 (4)	0.2777 (3)	0.12324 (11)	0.0591 (5)	
C11	0.7909 (5)	0.1680 (3)	0.33913 (12)	0.0621 (5)	
H11	0.747162	0.131003	0.297010	0.074*	
C6	−0.1322 (4)	0.4180 (3)	0.13258 (12)	0.0620 (5)	
C13	1.0487 (5)	0.1457 (3)	0.43582 (13)	0.0693 (6)	
H13	1.178385	0.095483	0.458888	0.083*	
C12	0.9801 (5)	0.0925 (3)	0.37353 (13)	0.0690 (6)	
H12	1.062501	0.004788	0.354580	0.083*	
C15	0.7318 (5)	0.3509 (3)	0.42988 (11)	0.0676 (6)	
H15	0.648243	0.437172	0.449493	0.081*	
C14	0.9221 (5)	0.2750 (3)	0.46366 (13)	0.0735 (7)	
H14	0.966483	0.310908	0.505855	0.088*	
C16	0.5102 (5)	0.7581 (3)	0.39759 (14)	0.0755 (7)	
H16A	0.429300	0.821692	0.365563	0.113*	
H16B	0.689782	0.743522	0.387050	0.113*	
H16C	0.507312	0.815778	0.438727	0.113*	
C18	−0.2471 (6)	0.1157 (4)	0.04230 (15)	0.0884 (9)	
H18A	−0.223724	0.046870	0.004568	0.133*	
H18B	−0.356737	0.203034	0.031583	0.133*	
H18C	−0.329956	0.049683	0.074221	0.133*	
C19	0.2318 (8)	−0.0502 (3)	0.1815 (2)	0.1081 (11)	
H19A	0.058829	−0.073200	0.161721	0.162*	
H19B	0.218626	−0.043294	0.227342	0.162*	
H19C	0.341133	−0.137746	0.169734	0.162*	
C17	−0.2436 (9)	0.6092 (4)	0.05619 (19)	0.1199 (14)	
H17A	−0.331407	0.608230	0.014676	0.180*	
H17B	−0.056050	0.624212	0.051646	0.180*	
H17C	−0.300412	0.698631	0.082812	0.180*	

### Atomic displacement parameters (Å^2^)

**Table:**

	*U*^11^	*U*^22^	*U*^33^	*U*^12^	*U*^13^	*U*^23^
O1	0.0628 (8)	0.0517 (7)	0.0575 (8)	0.0094 (6)	−0.0003 (7)	−0.0097 (6)
O2	0.0782 (10)	0.0584 (8)	0.0601 (9)	0.0080 (7)	0.0036 (8)	−0.0145 (7)
O7	0.0779 (10)	0.0586 (9)	0.0692 (10)	0.0145 (7)	0.0065 (8)	−0.0124 (7)
O3	0.0744 (10)	0.0578 (9)	0.0823 (11)	0.0181 (7)	0.0011 (8)	−0.0153 (8)
O4	0.0755 (10)	0.0626 (9)	0.0847 (12)	0.0200 (8)	−0.0046 (9)	−0.0056 (8)
O6	0.0745 (10)	0.0820 (11)	0.0589 (9)	0.0029 (8)	0.0009 (8)	−0.0155 (8)
O5	0.0868 (11)	0.0719 (10)	0.0773 (12)	0.0038 (9)	−0.0178 (9)	0.0043 (9)
C9	0.0550 (10)	0.0496 (10)	0.0547 (11)	0.0038 (8)	0.0037 (9)	−0.0023 (8)
C2	0.0550 (10)	0.0492 (10)	0.0541 (11)	0.0013 (8)	0.0058 (9)	−0.0070 (8)
C1	0.0545 (10)	0.0497 (10)	0.0591 (12)	0.0011 (8)	0.0036 (9)	−0.0022 (9)
C3	0.0616 (11)	0.0486 (10)	0.0565 (12)	0.0016 (9)	0.0064 (9)	−0.0079 (8)
C10	0.0585 (11)	0.0474 (10)	0.0583 (12)	0.0025 (8)	0.0050 (9)	−0.0016 (9)
C8	0.0612 (11)	0.0510 (10)	0.0585 (12)	0.0047 (9)	0.0063 (9)	−0.0053 (9)
C4	0.0595 (11)	0.0472 (10)	0.0657 (13)	0.0040 (9)	0.0096 (10)	−0.0058 (9)
C5	0.0591 (11)	0.0503 (11)	0.0702 (14)	0.0062 (9)	0.0036 (10)	−0.0006 (10)
C7	0.0628 (12)	0.0570 (11)	0.0560 (12)	−0.0022 (9)	0.0039 (10)	−0.0046 (9)
C11	0.0675 (12)	0.0539 (11)	0.0639 (13)	0.0065 (10)	−0.0025 (10)	−0.0081 (9)
C6	0.0617 (12)	0.0585 (12)	0.0651 (13)	0.0012 (10)	−0.0036 (10)	0.0039 (10)
C13	0.0669 (13)	0.0645 (13)	0.0766 (16)	0.0085 (11)	−0.0065 (12)	0.0067 (11)
C12	0.0695 (13)	0.0565 (12)	0.0816 (16)	0.0148 (10)	−0.0023 (12)	−0.0039 (11)
C15	0.0737 (14)	0.0685 (13)	0.0608 (13)	0.0133 (11)	0.0015 (11)	−0.0085 (11)
C14	0.0782 (15)	0.0806 (16)	0.0610 (14)	0.0112 (13)	−0.0066 (12)	−0.0056 (12)
C16	0.0753 (15)	0.0613 (13)	0.0871 (17)	0.0041 (11)	−0.0025 (13)	−0.0212 (12)
C18	0.0800 (16)	0.0908 (19)	0.090 (2)	−0.0031 (14)	−0.0024 (14)	−0.0297 (15)
C19	0.129 (3)	0.0560 (15)	0.142 (3)	0.0176 (16)	0.020 (2)	0.0052 (17)
C17	0.164 (4)	0.085 (2)	0.107 (3)	−0.005 (2)	−0.047 (3)	0.0312 (19)

### Geometric parameters (Å, º)

**Table:**

O1—C9	1.360 (3)	C5—C6	1.387 (3)
O1—C2	1.368 (2)	C7—C6	1.414 (3)
O2—C3	1.376 (2)	C11—C12	1.374 (3)
O2—C16	1.434 (3)	C11—H11	0.9300
O7—C8	1.373 (3)	C13—C12	1.376 (4)
O7—C19	1.413 (3)	C13—C14	1.384 (4)
O3—C4	1.252 (3)	C13—H13	0.9300
O4—C5	1.357 (3)	C12—H12	0.9300
O4—H4	0.8200	C15—C14	1.373 (4)
O6—C7	1.365 (3)	C15—H15	0.9300
O6—C18	1.415 (3)	C14—H14	0.9300
O5—C6	1.372 (3)	C16—H16A	0.9600
O5—C17	1.416 (4)	C16—H16B	0.9600
C9—C8	1.386 (3)	C16—H16C	0.9600
C9—C10	1.393 (3)	C18—H18A	0.9600
C2—C3	1.370 (3)	C18—H18B	0.9600
C2—C1	1.474 (3)	C18—H18C	0.9600
C1—C15	1.391 (3)	C19—H19A	0.9600
C1—C11	1.403 (3)	C19—H19B	0.9600
C3—C4	1.444 (3)	C19—H19C	0.9600
C10—C5	1.406 (3)	C17—H17A	0.9600
C10—C4	1.443 (3)	C17—H17B	0.9600
C8—C7	1.390 (3)	C17—H17C	0.9600
			
C9—O1—C2	121.52 (16)	C5—C6—C7	119.4 (2)
C3—O2—C16	114.26 (18)	C12—C13—C14	119.1 (2)
C8—O7—C19	114.8 (2)	C12—C13—H13	120.4
C5—O4—H4	109.5	C14—C13—H13	120.4
C7—O6—C18	119.0 (2)	C11—C12—C13	120.5 (2)
C6—O5—C17	115.0 (2)	C11—C12—H12	119.7
O1—C9—C8	116.32 (18)	C13—C12—H12	119.7
O1—C9—C10	121.69 (19)	C14—C15—C1	120.7 (2)
C8—C9—C10	122.0 (2)	C14—C15—H15	119.7
O1—C2—C3	119.82 (19)	C1—C15—H15	119.7
O1—C2—C1	110.68 (17)	C15—C14—C13	120.9 (2)
C3—C2—C1	129.50 (19)	C15—C14—H14	119.5
C15—C1—C11	117.9 (2)	C13—C14—H14	119.5
C15—C1—C2	123.54 (19)	O2—C16—H16A	109.5
C11—C1—C2	118.60 (19)	O2—C16—H16B	109.5
C2—C3—O2	120.3 (2)	H16A—C16—H16B	109.5
C2—C3—C4	121.67 (19)	O2—C16—H16C	109.5
O2—C3—C4	117.84 (18)	H16A—C16—H16C	109.5
C9—C10—C5	118.9 (2)	H16B—C16—H16C	109.5
C9—C10—C4	119.0 (2)	O6—C18—H18A	109.5
C5—C10—C4	122.2 (2)	O6—C18—H18B	109.5
O7—C8—C9	120.3 (2)	H18A—C18—H18B	109.5
O7—C8—C7	121.05 (19)	O6—C18—H18C	109.5
C9—C8—C7	118.6 (2)	H18A—C18—H18C	109.5
O3—C4—C10	121.9 (2)	H18B—C18—H18C	109.5
O3—C4—C3	121.7 (2)	O7—C19—H19A	109.5
C10—C4—C3	116.34 (18)	O7—C19—H19B	109.5
O4—C5—C6	119.3 (2)	H19A—C19—H19B	109.5
O4—C5—C10	120.4 (2)	O7—C19—H19C	109.5
C6—C5—C10	120.2 (2)	H19A—C19—H19C	109.5
O6—C7—C8	117.0 (2)	H19B—C19—H19C	109.5
O6—C7—C6	122.1 (2)	O5—C17—H17A	109.5
C8—C7—C6	120.8 (2)	O5—C17—H17B	109.5
C12—C11—C1	120.9 (2)	H17A—C17—H17B	109.5
C12—C11—H11	119.5	O5—C17—H17C	109.5
C1—C11—H11	119.5	H17A—C17—H17C	109.5
O5—C6—C5	121.6 (2)	H17B—C17—H17C	109.5
O5—C6—C7	119.0 (2)		
			
C2—O1—C9—C8	−179.93 (18)	C2—C3—C4—C10	−0.5 (3)
C2—O1—C9—C10	−0.8 (3)	O2—C3—C4—C10	−176.28 (18)
C9—O1—C2—C3	0.1 (3)	C9—C10—C5—O4	177.6 (2)
C9—O1—C2—C1	179.66 (17)	C4—C10—C5—O4	−1.6 (3)
O1—C2—C1—C15	−172.2 (2)	C9—C10—C5—C6	−3.7 (3)
C3—C2—C1—C15	7.3 (4)	C4—C10—C5—C6	177.1 (2)
O1—C2—C1—C11	7.3 (3)	C18—O6—C7—C8	128.1 (3)
C3—C2—C1—C11	−173.2 (2)	C18—O6—C7—C6	−56.3 (3)
O1—C2—C3—O2	176.21 (17)	O7—C8—C7—O6	−2.2 (3)
C1—C2—C3—O2	−3.2 (3)	C9—C8—C7—O6	174.70 (19)
O1—C2—C3—C4	0.6 (3)	O7—C8—C7—C6	−177.85 (19)
C1—C2—C3—C4	−178.9 (2)	C9—C8—C7—C6	−1.0 (3)
C16—O2—C3—C2	109.3 (2)	C15—C1—C11—C12	−0.5 (3)
C16—O2—C3—C4	−74.9 (2)	C2—C1—C11—C12	180.0 (2)
O1—C9—C10—C5	−178.38 (18)	C17—O5—C6—C5	66.7 (4)
C8—C9—C10—C5	0.7 (3)	C17—O5—C6—C7	−115.1 (3)
O1—C9—C10—C4	0.8 (3)	O4—C5—C6—O5	1.2 (4)
C8—C9—C10—C4	179.88 (19)	C10—C5—C6—O5	−177.50 (19)
C19—O7—C8—C9	91.8 (3)	O4—C5—C6—C7	−177.0 (2)
C19—O7—C8—C7	−91.4 (3)	C10—C5—C6—C7	4.4 (3)
O1—C9—C8—O7	−2.4 (3)	O6—C7—C6—O5	4.3 (3)
C10—C9—C8—O7	178.53 (19)	C8—C7—C6—O5	179.8 (2)
O1—C9—C8—C7	−179.25 (18)	O6—C7—C6—C5	−177.5 (2)
C10—C9—C8—C7	1.7 (3)	C8—C7—C6—C5	−2.0 (3)
C9—C10—C4—O3	179.0 (2)	C1—C11—C12—C13	−0.3 (4)
C5—C10—C4—O3	−1.8 (3)	C14—C13—C12—C11	0.8 (4)
C9—C10—C4—C3	−0.2 (3)	C11—C1—C15—C14	0.8 (4)
C5—C10—C4—C3	179.03 (19)	C2—C1—C15—C14	−179.7 (2)
C2—C3—C4—O3	−179.7 (2)	C1—C15—C14—C13	−0.3 (4)
O2—C3—C4—O3	4.6 (3)	C12—C13—C14—C15	−0.5 (4)

### Hydrogen-bond geometry (Å, º)

**Table:**

*D*—H···*A*	*D*—H	H···*A*	*D*···*A*	*D*—H···*A*
O4—H4···O3	0.82	1.87	2.599 (2)	147
C16—H16*A*···O3	0.96	2.51	3.079 (3)	118
C16—H16*B*···O3^i^	0.96	2.39	3.258 (3)	150
C18—H18*B*···O5	0.96	2.28	2.897 (4)	121
C18—H18*C*···O7^ii^	0.96	2.53	3.278 (4)	135
C17—H17*C*···O4	0.96	2.52	3.010 (4)	111

Symmetry codes: (i) *x*+1, *y*, *z*; (ii) *x*−1, *y*, *z*.
